# Phylogenetic Position of Aquificales Based on the Whole Genome Sequences of Six Aquificales Species

**DOI:** 10.1155/2012/859264

**Published:** 2012-07-12

**Authors:** Kenro Oshima, Yoko Chiba, Yasuo Igarashi, Hiroyuki Arai, Masaharu Ishii

**Affiliations:** ^1^Department of Agricultural and Environmental Biology, Graduate School of Agricultural and Life Sciences, The University of Tokyo, Bunkyo-ku, Tokyo 113-8657, Japan; ^2^Department of Biotechnology, Graduate School of Agricultural and Life Sciences, The University of Tokyo, Bunkyo-ku, Tokyo 113-8657, Japan

## Abstract

Species belonging to the order Aquificales are believed to be an early branching lineage within the Bacteria. However, the branching order of this group in single-gene phylogenetic trees is highly variable; for example, it has also been proposed that the Aquificales should be grouped with **ε**-proteobacteria. To investigate the phylogenetic position of Aquificales at the whole-genome level, here we reconstructed the phylogenetic trees of 18 bacteria including six Aquificales species based on the concatenated data of proteins shared by these bacteria. In the phylogenetic tree based on the whole-genome information, Aquificales was more closely related to Thermotogales than to Proteobacteria, suggesting that the Aquificales is a relatively early branching lineage within the Bacteria. Moreover, we classified the phylogenetic tree of each conserved orthologous protein by its topology. As a result, in the most major type of the phylogenetic trees, Aquificales was closely related to the Thermotogales. However, Aquificales was closely related to **ε**-proteobacteria in 21.0% of all phylogenetic trees, suggesting that many proteins phylogenetically related to the **ε**-proteobacteria may be encoded in the genomes of the members of the Aquificales. This unique feature may be responsible for the high variability in the branching order of Aquificales in single-gene phylogenetic trees.

## 1. Introduction

Species belonging to the order Aquificales are non-spore-forming, Gram-negative rods that are strictly thermophilic with optimal growth usually occurring above 65°C [1–3]. In terms of metabolism, most species of Aquificales are hydrogen-oxidizing bacteria that utilize hydrogen as the sole electron donor and oxygen as the electron acceptor [4]. Alternatively, thiosulfate or sulfur can also be used as a source of energy. Because of their thermostability, many enzymes found in this group are of interest for industrial and biotechnological applications [5].

Presently, the Aquificales species are believed to be the earliest branching lineage within the Bacteria [6–10]. However, the branching order of this group in single-gene phylogenetic trees is highly variable, and the deep branching of Aquificales is not supported by many protein phylogenies. For example, *Aquifex* has been shown to be close to *ε*-proteobacteria [11, 12] or the Chlamydiae group [6] in many protein phylogenies. Conserved inserts and deletions in a number of different proteins also provide evidence that the Aquificales is a late branching group within the Bacteria [13]. Many of these analyses suggest that Aquificales might be more closely related to Proteobacteria than to Thermotogales. Therefore, it is important to understand the phylogenetic position of Aquificales within the bacterial phylogeny.

The phylogenetic tree based on 16S rRNA sequences provides the presently accepted framework for understanding the evolutionary relationships among bacteria [14]. However, phylogenetic analysis at the single-gene level may provide only a limited understanding of the relationships and evolutionary history of bacteria, especially the closely related species that diverged at almost the same time [15]. In addition, species phylogenies derived from comparisons of different genes do not always concur, which may be attributed to lateral gene transfer [16], saturation with respect to amino acid substitutions [17], or highly variable rates of evolution of individual genes [18, 19]. Therefore, it is believed that comparative studies based on the complete sequences of bacterial genomes should form the basis for phylogeny and, ultimately, taxonomy [20].

The phylogenies inferred from concatenated data of housekeeping proteins amplified the resolving power for delineating the phylogenetic relationships among prokaryotes [21–23]. The complete genome of *Aquifex aeolicus* was sequenced in 1998 [3], and the genomes of five Aquificales species (*Hydrogenobacter thermophilus* TK-6, *Hydrogenobaculum* sp. Y04AAS1, *Persephonella marina*, *Sulfurihydrogenibium azorense* and *Sulfurihydrogenibium* sp. YO3AOP1) have recently been sequenced [24, 25]. Here we reconstructed the phylogenetic trees of 18 bacteria including six Aquificales bacteria based on the concatenated data of proteins shared by these bacteria. Moreover, the phylogenetic relationship between Aquificales and *ε*-proteobaceria was analyzed at the whole genome level.

## 2. Materials and Methods

In this study, we used 18 genome sequences from *Aquifex aeolicus*, *Hydrogenobacter thermophilus* TK-6, *Hydrogenobaculum* sp. Y04AAS1, *Persephonella marina*, *Sulfurihydrogenibium azorense*, *Sulfurihydrogenibium* sp. YO3AOP1, *Bacillus subtilis* subsp. *subtilis* str. 168, *Burkholderia mallei* ATCC 23344, *Campylobacter jejuni* subsp. *jejuni* NCTC 11168, *Chlamydophila pneumoniae* CWL029, *Deinococcus radiodurans* R1, *Thermus thermophilus* HB8, *Escherichia coli* str. K-12 substr. MG1655, *Salmonella enterica* subsp. *enterica* serovar Typhimurium LT2, *Helicobacter pylori* 26695, *Pyrococcus horikoshii* OT3, *Thermotoga maritima* MSB8 and *Thermotoga petrophila* RKU-1. These genome sequences was obtained from GenomeNet (http://www.genome.jp/).

First, BLASTP searches (each protein encoded in the genome of *Hydrogenobacter thermophilus* TK-6 was used as a query) were performed against 18 whole genomes by using stand-alone BLAST program [26]. If 18 different proteins from all 18 bacteria occupied the top 18 proteins of the result of the BLAST search, additional BLASTP searches were performed against 18 whole genomes by using each of top 18 proteins as a query. If the top 18 proteins in all 18 BLAST search are the same, we defined these 18 proteins as a conserved orthologous protein. This procedure enabled us to define 62 sets of orthologous proteins from the 18 genomes in our study. (see Supplementary Table 1 in supplementary material available online at doi:10.1155/2012/859264).

Next, we constructed 62 multiple-alignments using MUSCLE [27]. After that, a concatenated multiple alignment inferred from the 62 multiple alignments was generated. The concatenated alignment had 31,542 amino acid sites, including 15,442 gap/insertion sites that were not considered in this analysis. To avoid a potential cause for long branch attraction, we removed the most saturated sites from the whole multiple alignments according to the previously described method by Boussau et al. [28] as follows. First, PhyML [29] was used to build a starting phylogeny based on the whole multiple alignments, using the JTT model and a gamma law discretized in four classes to account for variation in the evolutionary rates. Second, to estimate how sites were modeled by the discretized gamma law, we plotted the distribution of expected relative evolutionary rates across sites as found by BppML (Supplementary Figure 1). Third, to reduce risks of long branch attraction, we decided to discard sites whose evolutionary rate was above the threshold of 2.0 (red line, Supplementary Figure 1). Finally, phylogenetic analyses were performed based on 10,000 amino acid sites. Based on the multiple alignments, a maximum likelihood (ML) tree was reconstructed using the PhyML [29] based on the JTT model and a gamma law discretized in four classes to account for variation in the evolutionary rates. *Pyrococcus horikoshii* was used as an outgroup. The confidence values (%) were estimated with the bootstrap sampling method (200 replications).

In addition, to reduce the influence of compositional bias, we recoded the alignment without saturated sites in 4 states based on the physicochemical properties of the amino acids [28] as follows: aromatic (FWY) and hydrophobic (MILV) amino acids were grouped in a single state, basic amino-acids (HKR) in another, acidic (DENQ) amino acids in one more state, and the fourth state contained all other amino acids (AGPST) to the exception of cysteine which was coded as missing data. The ML tree was constructed with this recoded alignment by the GTR model, an estimated proportion of invariant sites, a gamma law discretized in 5 categories with its alpha parameter estimated, and 200 bootstrap replicates [28].

To construct the phylogenetic tree of six Aquificales species, two Thermotogales species, two *γ*-proteobacteria, and two *ε*-proteobacteria, we used 12 genome sequences from *Aquifex aeolicus*, *Hydrogenobacter thermophilus* TK-6, *Hydrogenobaculum* sp. Y04AAS1, *Persephonella marina*, *Sulfurihydrogenibium azorense*, *Sulfurihydrogenibium* sp. YO3AOP1, *Campylobacter jejuni *subsp. *jejuni* NCTC 11168, *Deinococcus radiodurans *R1, *Escherichia coli* str. K-12 substr. MG1655, *Salmonella enterica *subsp. *enterica* serovar Typhimurium LT2, and *Helicobacter pylori *26695.

To construct the phylogenetic tree of Thermales-Deinococcales species, Thermotogales species, *γ*-proteobacteria, and *ε*-proteobacteria, we used 8 genome sequences from *Campylobacter jejuni* subsp. *jejuni* NCTC 11168, *Deinococcus radiodurans* R1, *Thermus thermophilus* HB8, *Escherichia coli *str. K-12 substr. MG1655, *Salmonella enterica *subsp. *enterica* serovar Typhimurium LT2, *Helicobacter pylori *26695, *Thermotoga maritima *MSB8, and *Thermotoga petrophila *RKU-1. The phylogenetic tree based on the concatenate data of the whole conserved orthologous proteins was constructed by the same method as described above. The ML trees of individual proteins were constructed using PhyML [28].

## 3. Results and Discussion

### 3.1. Phylogenetic Position of Aquificales Based on Whole-Genome Sequences

**Figure 1:**
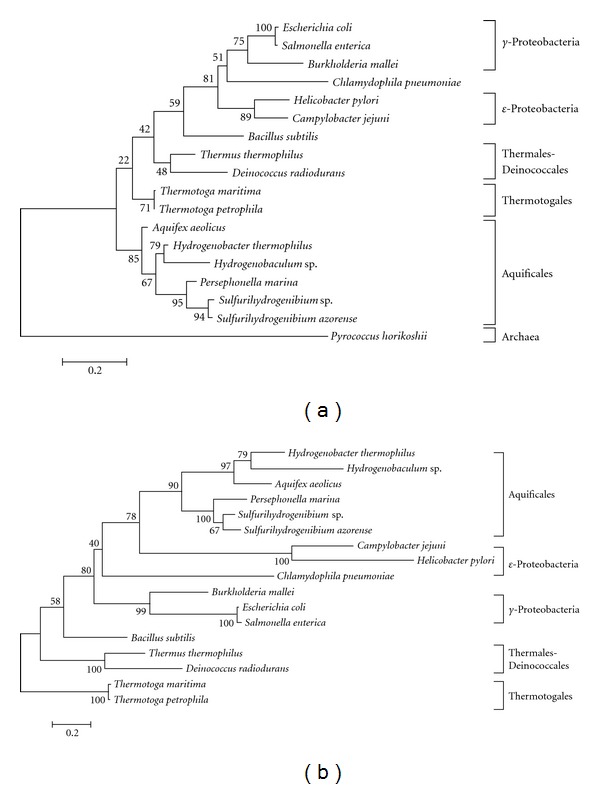
(a) Maximum likelihood tree based on the 16S rRNA sequence comparison. The number at each node represents the percentage in the bootstrap analysis (1000 replicates). (b) Maximum likelihood tree based on the amino acid sequence of the transcription elongation factor. The number at each node represents the percentage in the bootstrap analysis (1000 replicates).

First, we constructed ML trees based on the 16S rRNA sequences of 18 bacteria (Figure 1(a)). This phylogenetic tree indicated that each bacteria belonging to Archaea, Aquificales, Thermales-Deinococcales, and Thermotogales was clustered as a clade. Thermales-Deinococcales was clustered with proteobacteria with 74% bootstrap support. The closest species to Archaea was the bacteria belonging to Thermotogales, and the second nearest neighbor was Aquificales, suggesting that the Aquificales species are an early branching lineage within the Bacteria. In contrast, the topology of the phylogenetic tree based on the amino acid sequences of transcription elongation factor (NusA) (Figure 1(b)) differed from that of the 16S rRNA gene. For example, Aquificales species were clustered with *ε*-proteobacteria with 80% bootstrap support, suggesting that the Aquificales is a late branching group within the Bacteria.

**Figure 2:**
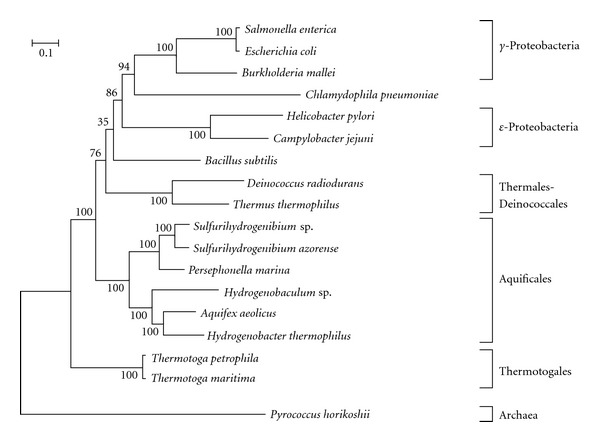
Maximum likelihood tree based on the comparison of 62 proteins; 10,000 amino acid sites were considered (see Section 2). The number at each node represents the percentage in the bootstrap analysis (200 replicates).

To investigate the phylogenetic position of Aquificales species at the whole-genome level, we constructed a phylogenetic tree based on 18 whole genomes of Archaea, Aquificales, Thermales-Deinococcales, Thermotogales, and related bacteria. First, 62 orthologous gene families that are shared by all 18 bacteria were selected (Supplementary Table  1). To avoid a potential cause for long branch attraction, we removed the most saturated sites from the whole multiple alignments according to the previously described method by Boussau et al. [28]. As a result, 10,000 amino acid sites were considered in the maximum likelihood analysis. The phylogenetic tree based on the whole-genome information indicated that the 18 bacteria were divided into six major groups (Archaea, Aquificales, Thermotogales, Thermales-Deinococcales, *γ*-proteobacteria, and *ε*-proteobacteria) with 100% bootstrap support. Analysis of signature sequences (consisting of conserved inserts or deletions) in highly conserved proteins suggested that the Aquificales diverged after the branching of Thermotogales, Thermales-Deinococcales, Cyanobacteria, Spirochetes, and Chlamydiae, but before the emergence of Proteobacteria [13]. However, in the phylogenetic tree based on the whole-genome information, the Archaea group was evolutionarily closely related to the Thermotogales, and Aquificales was a neighbor to Thermotogales with 76% bootstrap value (Figure 2). These analyses suggest that Aquificales is more closely related to Thermotogales than to Proteobacteria, which is consistent with the phylogenetic relationship showed by Boussau et al. [28]. To reduce the influence of compositional bias, we recoded the concatenated protein alignment in 4 states based on the physicochemical properties of the amino acids, and constructed a phylogenetic tree. As a result, although *Bacillus subtilis* was clustered with Thermales-Deinococcales, the ML tree obtained by the recoded alignment (Supplemetary Figure  2) was very similar to the previous tree (Figure 2), implying that the Aquificales-Thermotogales grouping does not seem to result from compositional biases. These results suggest that the Aquificales species are a relatively early branching lineage within the Bacteria.

### 3.2. Phylogenetic Relationships between the Aquificales and *ε*-Proteobacteria

**Figure 3:**
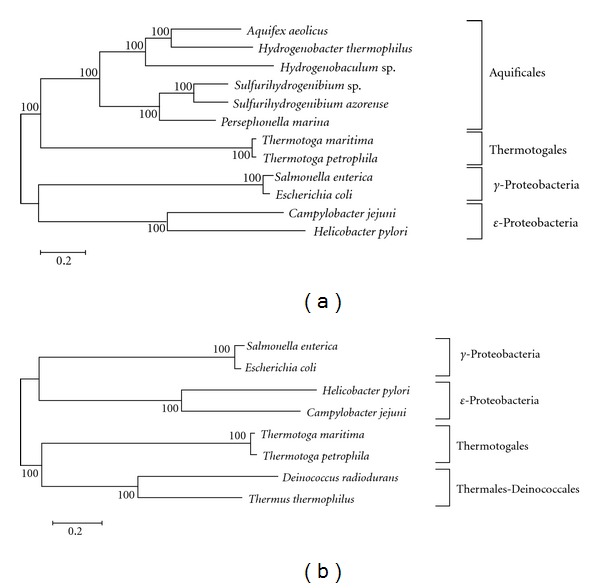
Unrooted maximum likelihood tree based on whole-genome information by using (a) the 271 conserved proteins among Aquificales, Thermotogales, *γ*-proteobacteria and *ε*-proteobacteria, or (b) the 259 conserved proteins among Thermales, Thermotogales, *γ*-proteobacteria and *ε*-proteobacteria. The number at each node represents the percentage in the bootstrap analysis (200 replicates).

It has been proposed that the Aquificales should be grouped with the *ε*-proteobacteria [12], which is supported by the phylogenetic analysis of single protein sequences such as the transcription elongation factor (Figure 1(b)). However, the late branching of the Aquificales is not supported by the 16S rRNA gene sequence tree (Figure 1(a)) and the phylogenetic tree based on the whole-genome information (Figure 2). To investigate the phylogenetic relationships between the Aquificales and *ε*-proteobacteria, we reconstructed phylogenetic trees of 12 bacteria including six Aquificales species, two Thermotogales species, two *γ*-proteobacteria, and two *ε*-proteobacteria based on the concatenated data of proteins shared by these bacteria. First, 271 orthologous gene families that are shared by all 12 bacteria were selected. As a result, 16,532 amino acid sites were considered in the ML analysis. The phylogenetic tree based on this whole-genome information indicated that the 12 bacteria were divided into four major groups (Aquificales, Thermotogales, *γ*-proteobacteria, and *ε*-proteobacteria) with 100% bootstrap support (Figure 3(a)). In addition, the phylogenetic tree based on the whole-genome information indicated that the Aquificales group was clustered with the Thermotogales group with 100% bootstrap support (Figure 3(a)). 

**Figure 4:**
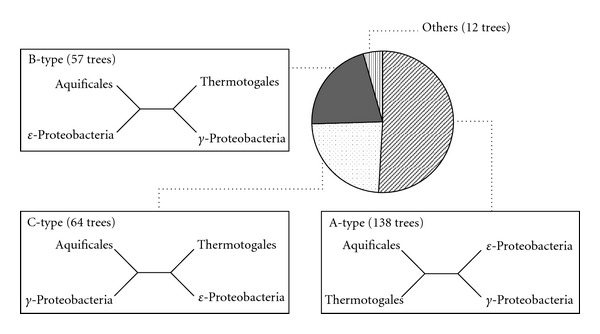
Distribution of topology of the phylogenetic trees of the 271 conserved proteins among Aquificales, Thermotogales, *γ*-proteobacteria, and *ε*-proteobacteria.

Next, to investigate the contribution of each protein to the whole-genome phylogenetic tree, we constructed 271 ML trees from 271 protein sets. We classified these trees into the following three types (Figure 4): A-type, the Aquificales group is more closely related to the Thermotogales group; B-type, the Aquificales group is more closely related to the *ε*-proteobacteria group; C-type, the Aquificales group is more closely related to the *γ*-proteobacteria group. The most frequent type of these phylogenetic trees was A-type (138 trees), which is consistent with the results obtained from the phylogenetic tree based on the 271 conserved proteins (Figure 3(a)). Interestingly, B-type trees occupied 21.0% (57 trees) of all phylogenetic trees (Table 1). For example, the Aquificales was clustered with the *ε*-proteobacteria with 94% bootstrap support in the phylogenetic tree of DNA polymerase I (Supplementary Figure  3). These results suggest that many proteins phylogenetically related to the *ε*-proteobacteria may be encoded in the genomes of the members of the Aquificales order.

**Figure 5:**
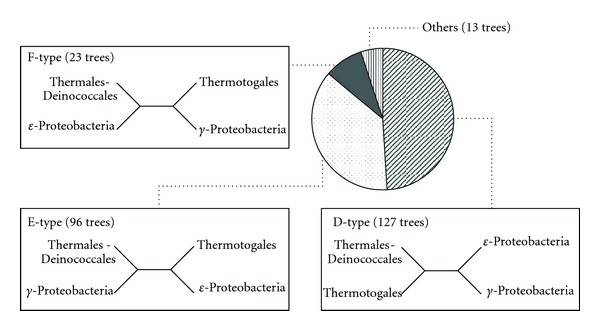
Distribution of topology of the phylogenetic trees of the 259 conserved proteins among Thermales, Thermotogales, *γ*-proteobacteria and *ε*-proteobacteria.

To compare this profile with that of other bacteria, we performed the same phylogenetic analysis against 259 conserved proteins among Thermales-Deinococcales, Thermotogales, *γ*-proteobacteria, and *ε*-proteobacteria. As a result, the Thermales-Deinococcales group was clustered with the Thermotogales group with 100% bootstrap support in the phylogenetic tree based on the whole genome conserved proteins (Figure 3(b)). Next, we classified the phylogenetic tree of each protein into the following three types (Figure 5); D-type, the Thermales-Deinococcales group is more closely related to the Thermotogales group; E-type, the Thermales-Deinococcales group is more closely related to the *γ*-proteobacteria group; F-type, the Thermales-Deinococcales group is more closely related to the *ε*-proteobacteria group. The most frequent type of these phylogenetic trees was D-type (127 trees), and E-type trees occupied 37.1% (96 trees) of all phylogenetic trees (Figure 5). In contrast, F-type trees occupied only 8.9% (23 trees) of all phylogenetic trees (Figure 5), suggesting that the phylogenetic relationship between the Thermales-Deinococcales and *ε*-proteobacteria may be low compared to the Aquificales. 

These results support the hypothesis that many proteins phylogenetically close to the *ε*-proteobacteria may be encoded in the genomes of the Aquificales. This unique feature may be responsible for the high variability in the branching order of Aquificales in single-gene phylogenetic trees. Moreover, these results raised the possibility that a large horizontal gene transfer had occurred between the Aquificales and *ε*-proteobacteria, which was suggested by Boussau et al. [28]. This hypothesis might be supported by the fact that *ε*-proteobacteria include hydrogen-oxidizing bacteria and sulfur-oxidizing bacteria [30] which occupy the same ecological niche with Aquificales. 

Several house-keeping proteins have often been used for the phylogenetic analyses of bacteria [31–33]. However, our results suggest that the phylogenetic position of single proteins is highly variable even for transcription elongation factor and DNA polymerase I. Therefore, whole-genome level phylogenetic approaches are extremely important and will possibly play a crucial role in the future studies of microbial evolution.

## Supplementary Material

List of 62 proteins to construct the phylogenetic tree based on 18 whole genomes

## Figures and Tables

**Table 1:** List of B-type conserved proteins that the Aquificales is clustered with *ε*-proteobacteria in the phylogenetic analysis (Figure 4). Accession numbers of conserved proteins of *Hydrogenobacterthermophilus* TK-6 are indicated.

Accession number	Putative function
YP_003431690	transcription elongation factor
YP_003432239	ribosomal protein S9
YP_003432379	ribosomal protein L18
YP_003432892	4-hydroxy-3-methylbut-2-enyl diphosphate reductase
YP_003432936	ATP-dependent protease
YP_003433556	UDP-N-acetylglucosamine pyrophosphorylase
YP_003431738	putative metalloprotease
YP_003431749	diaminopimelate decarboxylase
YP_003431809	dihydrodipicolinate reductase
YP_003431998	UDP-N-acetylglucosamine 1-carboxyvinyltransferase
YP_003432481	ribosomal protein S20
YP_003432953	queuine tRNA-ribosyltransferase
YP_003431834	ATP-dependent protease La
YP_003431839	tRNA delta(2)-isopentenylpyrophosphate transferase
YP_003431873	2-C-methyl-D-erythritol 4-phosphate cytidylyltransferase
YP_003431915	ribonuclease III
YP_003432036	riboflavin synthase alpha chain
YP_003432044	DNA polymerase I
YP_003432149	2-methylthioadenine synthetase
YP_003432165	folylpolyglutamate synthase
YP_003432232	DNA polymerase III beta subunit
YP_003432262	UDP-N-acetylglucosamine-N-acetylmuramyl- (pentapeptide) pyrophosphoryl-undecaprenol N-acetylglucosamine transferase
YP_003432385	methionine aminopeptidase
YP_003432408	methionyl-tRNA synthetase
YP_003432463	fatty acid/phospholipid synthesis protein
YP_003433015	carboxyl-terminal protease
YP_003433058	rRNA methylase
YP_003433377	3-phosphoshikimate 1-carboxyvinyltransferase
YP_003433542	arginyl-tRNA synthetase
YP_003431843	F0F1-type ATP synthase gamma subunit
YP_003431889	signal recognition particle GTPase
YP_003432507	ribosomal protein L22
YP_003432144	Holliday junction resolvase
YP_003432824	DNA processing protein
YP_003432257	GTP-binding protein
YP_003432274	triosephosphate isomerase
YP_003432330	aspartate 1-decarboxylase
YP_003432353	uridylate kinase
YP_003432374	ribosomal protein L24
YP_003432380	ribosomal protein S5
YP_003432524	transcription antitermination protein
YP_003432640	methionyl-tRNA formyltransferase
YP_003433333	ribosomal protein L20
YP_003432384	adenylate kinase
YP_003432390	ribosomal protein S4
YP_003432414	thiol peroxidase
YP_003432533	orotidine 5^′^-phosphate decarboxylase
YP_003432615	S-adenosyl-methyltransferase
YP_003432911	carbamoyl-phosphate synthase small subunit
YP_003432886	dihydrodipicolinate synthase
YP_003432967	membrane protein
YP_003432968	GMP synthase
YP_003433028	hypothetical protein HTH_1376
YP_003433124	homoserine kinase
YP_003433221	UDP-glucose-4-epimerase
YP_003433380	pantothenate metabolism flavoprotein
YP_003433549	cell cycle protein
