# Supplementary material for: Phylogenetic Position of Aquificales Based on the Whole Genome Sequences of Six Aquificales Species
Source: Int J Evol Biol. 2012 Jul 12;2012:859264. doi: 10.1155/2012/859264 (PMC3403428; doi:10.1155/2012/859264)
Supplement: Supplementary file 1 — List of 62 proteins to construct the phylogenetic tree based on 18 whole genomes [file 859264.f1.pdf]

Supplementary Table 1

List of 62 proteins to construct the phylogenetic tree based on 18 whole genomes (Fig. 2).

Accession number	Putative function
**YP_003431711.1**	phenylalanyl-tRNA synthetase beta chain
**YP_003431775.1**	3-phosphoglycerate kinase
**YP_003431873.1**	2-C-methyl-D-erythritol 4-phosphate cytidylyltransferase
**YP_003431886.1**	pseudouridylate synthase
**YP_003431889.1**	signal recognition particle GTPase
**YP_003431930.1**	acetyl-CoA carboxylase alpha subunit
**YP_003431961.1**	valyl-tRNA synthetase
**YP_003431972.1**	GTP-binding protein
**YP_003431987.1**	inosine-5'-monophosphate dehydrogenase
**YP_003432020.1**	aspartyl-tRNA synthetase
**YP_003432036.1**	riboflavin synthase alpha chain
**YP_003432147.1**	histidyl-tRNA synthetase
**YP_003432239.1**	ribosomal protein S9
**YP_003432240.1**	ribosomal protein L13
**YP_003432274.1**	triosephosphate isomerase
**YP_003432291.1**	methylenetetrahydrofolate dehydrogenase
**YP_003432353.1**	uridylate kinase
**YP_003432354.1**	translation elongation factor Ts
**YP_003432355.1**	ribosomal protein S2
**YP_003432373.1**	ribosomal protein L14
**YP_003432375.1**	ribosomal protein L5
**YP_003432377.1**	ribosomal protein S8
**YP_003432378.1**	ribosomal protein L6
**YP_003432380.1**	ribosomal protein S5
**YP_003432382.1**	ribosomal protein L15
**YP_003432383.1**	preprotein translocase SecY subunit
**YP_003432384.1**	adenylate kinase
**YP_003432388.1**	ribosomal protein S13
**YP_003432389.1**	ribosomal protein S11
**YP_003432390.1**	ribosomal protein S4
**YP_003432406.1**	dimethyladenosine transferase

**YP_003432408.1**	methionyl-tRNA synthetase
**YP_003432503.1**	ribosomal protein S17
**YP_003432506.1**	ribosomal protein S3
**YP_003432507.1**	ribosomal protein L22
**YP_003432508.1**	ribosomal protein S19
**YP_003432509.1**	ribosomal protein L2
**YP_003432512.1**	ribosomal protein L3
**YP_003432513.1**	ribosomal protein S10
**YP_003432518.1**	DNA-directed RNA polymerase beta' subunit
**YP_003432522.1**	ribosomal protein L1
**YP_003432523.1**	ribosomal protein L11
**YP_003432624.1**	dephospho-CoA kinase
**YP_003432637.1**	enolase
**YP_003432711.1**	cysteinyl-tRNA synthetase
**YP_003432747.1**	leucyl-tRNA synthetase
**YP_003432831.1**	polyribonucleotide nucleotidyltransferase
**YP_003432832.1**	ribosomal protein S15
**YP_003432924.1**	translation initiation factor IF-2
**YP_003432953.1**	queuine tRNA-ribosyltransferase
**YP_003432968.1**	GMP synthase
**YP_003433016.1**	O-sialoglycoprotein endopeptidase
**YP_003433062.1**	signal recognition particle
**YP_003433099.1**	ribonuclease HII
**YP_003433106.1**	CTP synthetase
**YP_003433243.1**	seryl-tRNA synthetase
**YP_003433320.1**	thymidylate kinase
**YP_003433334.1**	phenylalanyl-tRNA synthetase alpha subunit
**YP_003433441.1**	undecaprenyl pyrophosphate synthetase
**YP_003433497.1**	alanyl-tRNA synthetase
**YP_003433542.1**	arginyl-tRNA synthetase
**YP_003433549.1**	cell cycle protein
